# The Usefulness of Quantitative EEG and Advanced MR Techniques in the Monitoring and Long-Term Prognosis of Lance-Adams Syndrome

**DOI:** 10.3389/fneur.2019.00214

**Published:** 2019-03-12

**Authors:** Aleksandra Szczepańska, Edyta Dziadkowiak, Joanna Bladowska, Lech Kipiński, Sławomir Budrewicz, Magdalena Koszewicz

**Affiliations:** ^1^Department of Neurology, Wroclaw Medical University, Wroclaw, Poland; ^2^Department of General Radiology, Interventional Radiology and Neuroradiology, Wroclaw Medical University, Wroclaw, Poland; ^3^Department of Pathophysiology, Wroclaw Medical University, Wroclaw, Poland

**Keywords:** chronic post-hypoxic myoclonus, Lance-Adams syndrome, action myoclonus, quantitative EEG, MR spectroscopy

## Abstract

**Objectives:** Chronic post-hypoxic myoclonus, known as Lance-Adams syndrome (LAS), is a rare complication of successful cardiopulmonary resuscitation. It is characterized by intention myoclonus, cerebellar ataxia, and preserved intellect. The basis of the disease and its long-term prognosis remain unclear.

**Case report:** The authors present a 53-year-old woman with a history of asthma bronchiale who suffered from myoclonus after hypoxic brain damage due to cardiac arrest. Advanced electrophysiological (quantitative EEG) and MR (MR spectroscopy) techniques were employed.

**Conclusions:** Over long-term observation the results suggested permanent synaptic rearrangements of the neuronal networks due to brain plasticity in the patient after the brain hypoxia.

## Background

Chronic post-hypoxic myoclonus (PHM), known as Lance-Adams syndrome (LAS), is a rare disorder seen in survivors of profound hypoxic episodes. It is characterized by action myoclonus that starts days to weeks after cardiorespiratory resuscitation (CPR). Myoclonic jerks are specifically triggered by action, startle, and tactile stimulation, and they usually disappear either with body and limb relaxation or during sleep. The anatomic and neurochemical bases of chronic PHM still remain unclear, as does its clinical prognosis. The accurate distinction between myoclonic status epilepticus and LAS is important because of their different therapeutic proceedings (1–6). The authors present an 18-month clinical follow up of an LAS case with the use of the advanced diagnostic techniques of EEG and MR.

## Case Presentation

A 53-year-old woman with a history of asthma bronchiale and chronic obstructive pulmonary disease had a cardiorespiratory arrest due to status asthmaticus. After successful cardiopulmonary resuscitation the normal sinus rhythm returned after 10 min. Tonic-clonic seizures, action myoclonus and clinical pyramidal syndrome developed. The patient's condition improved after 7 weeks and she was weaned from mechanical ventilation and referred to the neurological and then rehabilitation unit.

She was first admitted to our neurological department 9 months after the cardiac arrest. Neurological examinations revealed: the patient was fully conscious, attentive, and oriented; she had mild cognitive concentration deficits and echolalia, right lateral end-gaze nystagmus and central VII cranial nerve deficit. Her motor strength was 4/5 throughout muscle tone was markedly increased in the lower extremities with brisk deep reflexes, and a positive Rossolimo sign on both sides. She also had ataxia in both upper limbs without any sensory deficit. Intention myoclonic jerks were noted in the face, trunk, and upper and lower extremities.

She was partly able to feed herself, sit up, get up, and walk with the help of a walker and other persons.

**Figure 1:**
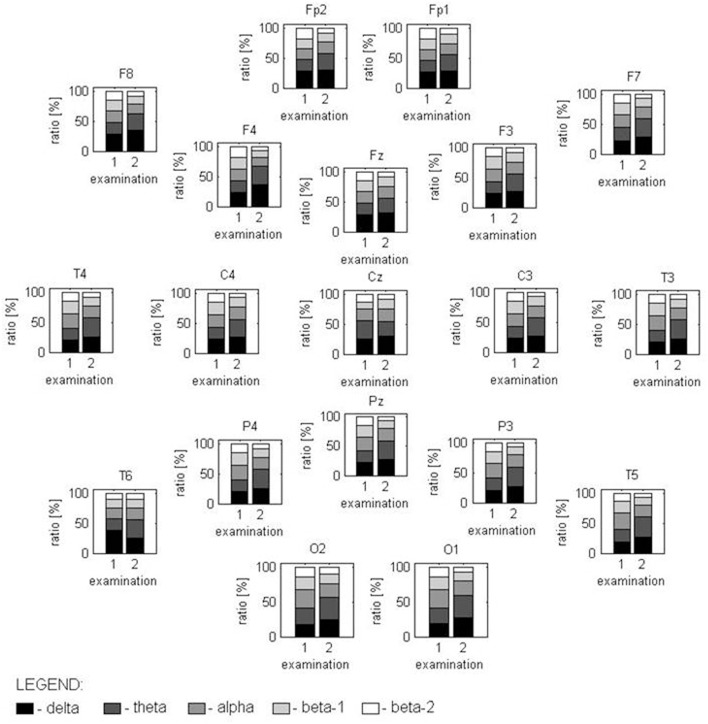
Distribution of the power relations between analyzed frequency bands, marked in gray scale, in both examinations for all EEG channels. In the first examination, spectral power with a statistically different distribution is found for the T6 channel (caused by high delta disturbances) and in the O1-02 channels (where the alpha activity is higher than in other locations). The second examination has a completely different spectral structure in all locations with the exception of Fz and Cz.

**Figure 2:**
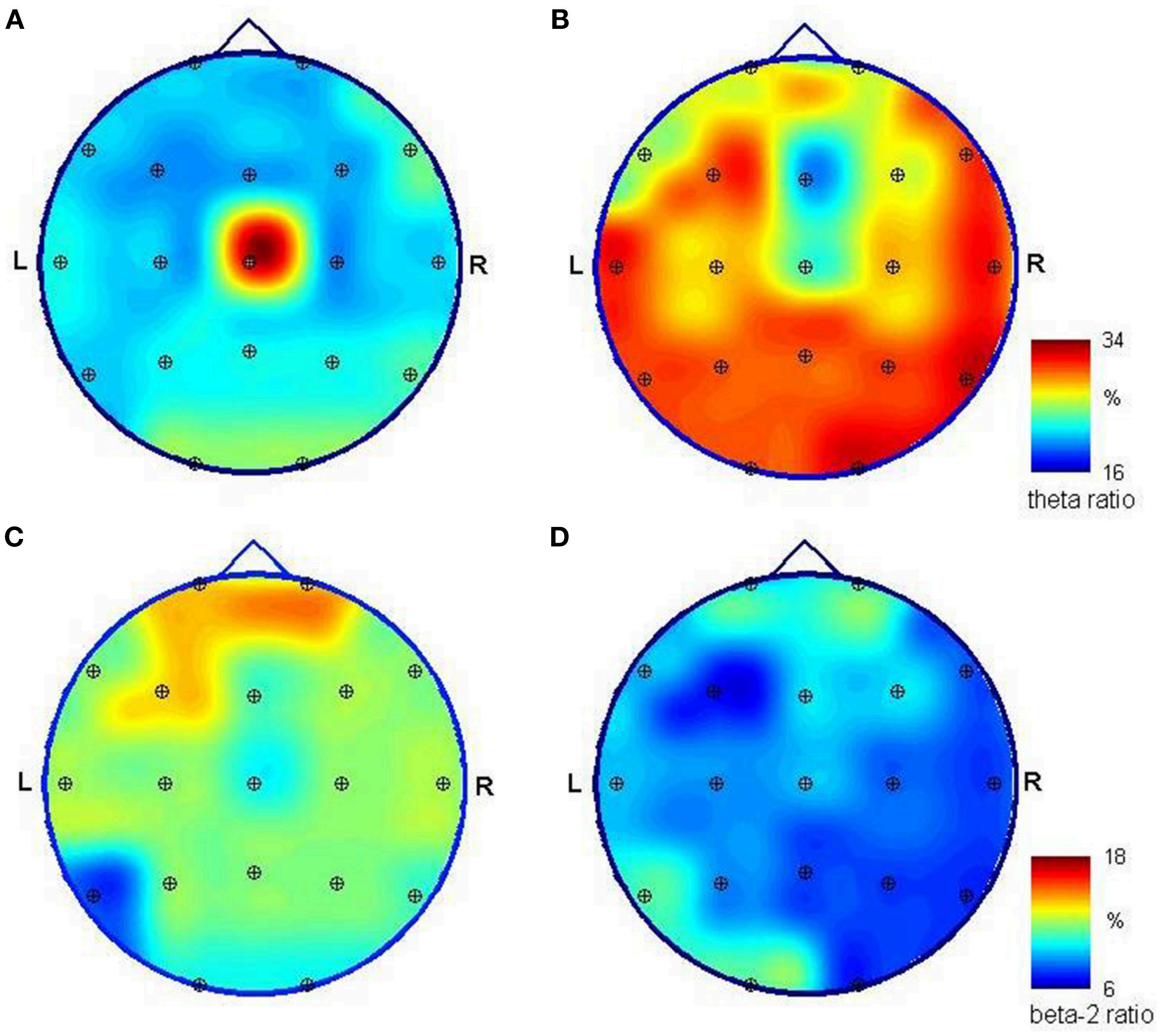
Mapping of the relative spectral power of EEG signals registered unipolar in reference to a frontal midline (Fp1/Fp2) relative spectral power of EEG signals registered during initial (left) and control (right) hospitalization. Spectra were obtained using FFT, and ratios were calculated for the theta (3.5–7.5 Hz) band **(A,B)** and beta-2 (18–24 Hz) band **(C,D)** as the power of EEG in a specific band divided by the level obtained for all bands (1–24 Hz). The high ration value obtained for theta activity in the first examination and the central location **(A)** is caused by the slow-frequency artifacts in the Cz channel. Theta activity dominates in posterior channels and is much stronger in both absolute and relative values in the second examination **(B)**. Fast beta activity is stronger in the frontal area; yet its relative power is much smaller in the second examination **(D)**, where its distribution has a predominance in the left hemisphere; however, the differences visible at **(C,D)** are not statistically significant.

Nine months after CPR, EEG showed correct basic activity and symmetric and synchronous paroxysmal sharp wave discharges and sharp and slow wave complexes on both sides. A hyperventilation test increased the number of discharges. Quantitative EEG (QEEG) analysis was performed in a frequency domain using the fast Fourier transform (FFT) algorithm following the guidelines given in Gupta and Caviness (4) and revealed a predominance of slow frequencies (1–7.5 Hz) measured as absolute spectral power (*p* < 0.01), with peaks at ~7.1 Hz in most of the 19 channels. On analyzing the ratios of relative power [in bands: delta (1.0–3.5 Hz), theta (3.5–7.5 Hz), alpha (7.5–12.5 Hz), beta-1 (12.5–18 Hz), and beta-2 (18–24 Hz)] as an amount of EEG activity in a frequency band divided by the amount in all bands, disproportions in spatial distribution (*p* < 0.05) were observed for both occipital channels (caused mainly by alpha activity) and the central area (electrodes Cz and Pz, caused by slow frequency predomination). We found no hemispherical asymmetry (*p* < 0.05) (Figures 1, 2A,C).

Hemispherical asymmetry was found in the multimodal evoked potentials. The latency of P100 of the visual evoked potential was bilaterally prolonged, more so on the right side (left P100 = 138 ms, right P100 = 146 ms). We also found a slight discrepancy between sides in inter-wave latencies III-V, I-V of the brainstem auditory evoked potentials, longer on the right side (2.32 and 4.57 ms vs. 1.94 and 4.12 ms). All somatosensory evoked potential parameters from the median nerve were within normal limits.

At the same time, we performed a brain MRI with a 1.5T unit (Signa Hdx, GE Medical System) using a 16-channel coil design for head and spine imaging. The imaging protocol included conventional axial, sagittal and coronal T2-weighted images, axial and coronal T1-weighted images and axial FLAIR (fluid-attenuated inversion recovery sequences) images, as well as MR spectroscopy (MRS). The MRS examinations were performed using the Single Voxel Spectroscopy (SVS) technique (PRESS sequence). Using localizing axial T2-weighted images, voxels of 2 × 2 × 2 cm (8 cm^3^) were placed in the posterior cingulate gyrus (PCG) and left parietal white matter (PWM). Ratios of N-acetylaspartate (NAA), choline (Cho) and myo-inositol (mI) to creatine (NAA/Cr, Cho/Cr, mI/Cr, respectively) were calculated and analyzed.

**Figure 3:**
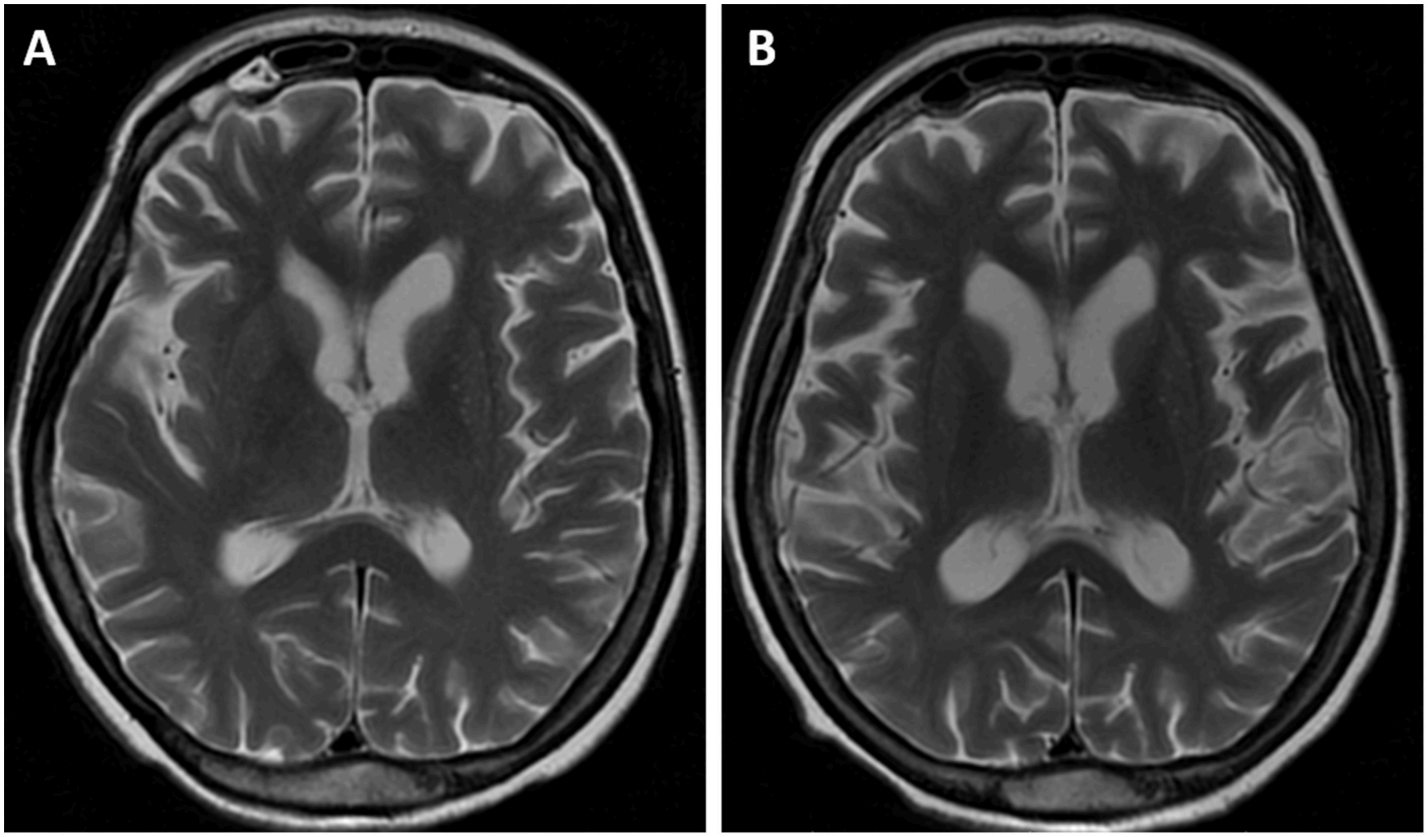
MR examination, axial T2-weighted images. The initial MR showed diffuse, mild cortico-subcortical atrophy of the brain **(A)**. The follow-up image **(B)** revealed an increased rate of brain atrophy after 2 years.

**Figure 4:**
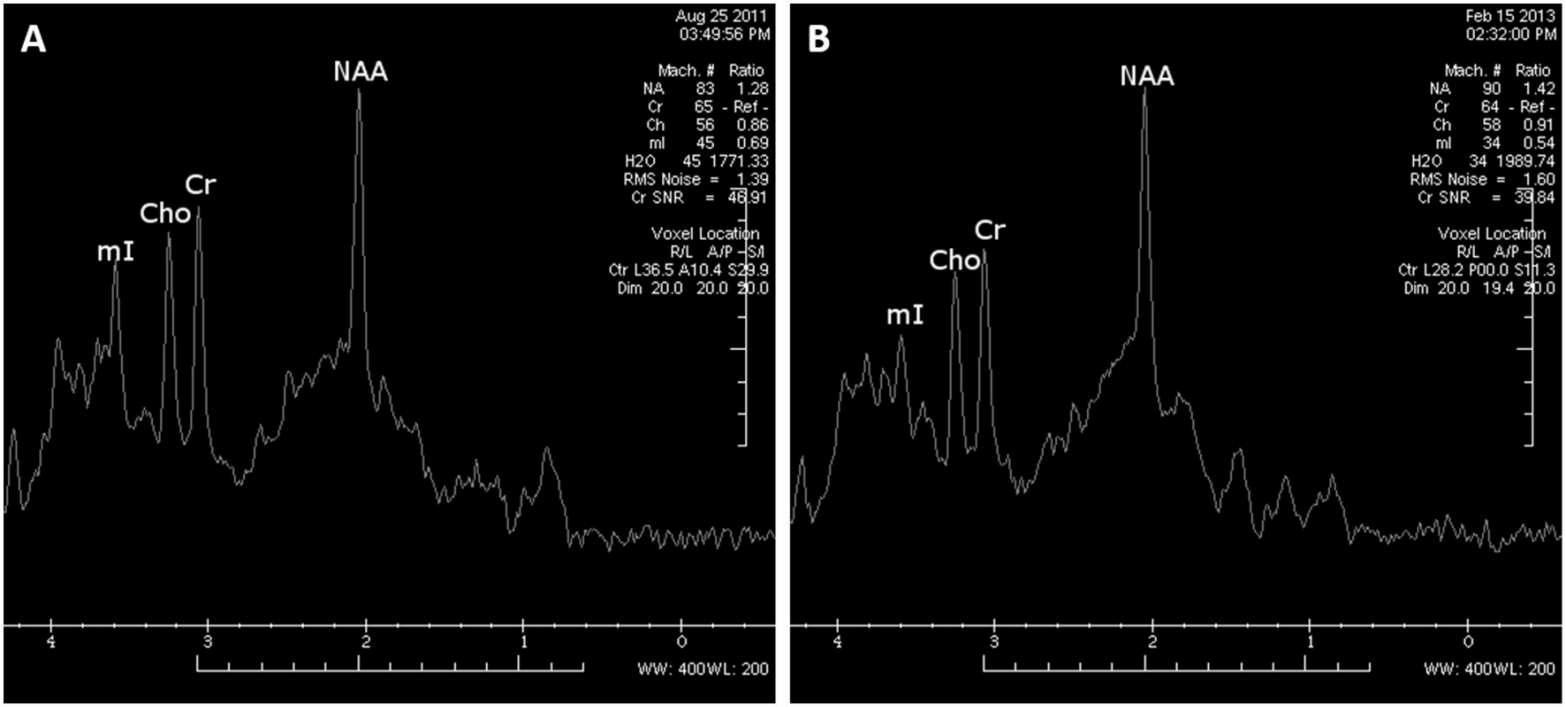
MRS examination within the left parietal white matter area performed at 9 months after cardiorespiratory arrest revealed decreased NAA/Cr ratios **(A)**. The control MRS examination (18 months after cardiorespiratory arrest) demonstrated a moderate improvement of NAA/Cr ratios within white matter; however, the NAA/Cr ratio still remained decreased **(B)**.

The MR images showed diffuse, mild cortico-subcortical brain atrophy (Figure 3A). MR spectroscopy revealed a decreased NAA/Cr ratio in the PCG region, as well as within the parietal white matter (Figure 4A); the NAA/Cr ratios were 1.25 and 1.28, respectively. The other metabolite ratios presented values within the normal limit, as follows: Cho/Cr = 0.53, mI/Cr = 0.57 in PCG and Cho/Cr = 0.86, mI/Cr = 0.69 in the PWM area.

No other abnormalities were noted. Laboratory results were within normal limits.

The patient received intensive rehabilitation and antiepileptic treatment (sodium valproate 2,000 mg/d, levetiracetam 1,000 mg/d).

Twenty six months after cardiac arrest the patient was admitted to our department for the second time because of an increased amount of myoclonic jerks. The patient presented a similar neurological status as before. She could do everyday activities, such as feeding or toilet on her own.

The levetiracetam dosage was increased (up to 3,000 mg/d) leading to a marked reduction in the myoclonus.

EEG performed 26 months after CPR showed the normal basic function of both hemispheres in comparison with the previous reduced results. The number and amplitude of slow wave discharges and sharp and slow wave complexes were reduced. QEEG revealed statistically significant (*p* < 0.05) differences in the proportions of the contribution of each frequency band in the spectrum between the current and previous examinations; proved for 17 of 19 EEG channels (with the exception of Fz and Cz). This is due to a change in the relative spectral power calculated for the 3.5–7 Hz band and (to a lesser extent) the 18–24 Hz band in the signal recorded 26 months after CPR in comparison to the EEG measured 17 months earlier (Figure 1). The first study presented a smaller relative-magnitude of theta and a larger one of beta-2 waves, and the ratio of energy possessed by EEG at both frequency bands (theta/beta-2) changed from 1.1, 1.7, and 1.4 to 3.4, 3.9, and 4.0 on average for frontal, occipital and tempo-cervical areas, respectively (*p* < 0.01). The dominant peak frequency in the theta band moved its position to ~6.2 Hz. No statistically significant disproportions were found in the spatial distribution of the relative spectral power. The spectral maps prepared for EEG recorded 9 and 26 months after CPR presented some changes in this distribution (Figure 2).

The follow-up MR image (Figure 3B) revealed an increased rate of brain atrophy after 2 years, MRS study—a slight improvement in NAA/Cr ratios in the cortex of PCG and a moderate one within white matter (Figure 4B), but still the NAA/Cr ratios remained decreased (NAA/Cr = 1.29 in PCG and NAA/Cr = 1.42 in PWM).

The patient gave her written informed consent to participate in the study, and for the publication of this case report.

## Discussion

LAS is a rare disorder. Diffuse brain damage was seen in the postmortem studies, but area, crucial for LAS development, remains unknown. Complex mechanisms with serotonin deficiency, permanent changes in the neuronal network, are probably involved in LAS generation (2, 4, 6). In the literature, EEG findings are variable, but often display bursts of generalized spikes and polyspikes or burst suppression patterns believed to be consistent with severe neuronal injury (7). In QEEG, we revealed a consolidation of the changes in the form of a slow activity over prolonged observation. In the literature, we did not find any other QEEG tests in the case of LAS. Conventional MR images may present only diffuse brain atrophy (8, 9), which was also seen in our case. Performing advanced MR techniques, such as MRS, it is possible to find far more cerebral alterations which are not visible in conventional MR. We observed a decreased NAA/Cr ratio in the apparently normal PCG cortex, as well as in parietal white matter regions. The reduction in the NAA/Cr ratio suggests a decrease in neuronal activity within the cortex of PCG and white matter, which may confirm hypoxic brain damage in the past. The electrophysiological findings of cortical myoclonus might be due to a reduction in the NAA/Cr ratio within the cortex of PCG (10). Zhang et al. (11) also reported a decreased NAA peak measured in both hippocampi in a woman after cardiorespiratory arrest.

Some neurochemical and neuroimaging evidence might highlight the functional and structural dynamic properties of the nervous system, particularly the cerebral cortex, indicating a non-linear neurological functional recovery after brain injury or ischemia. The improvement in NAA/Cr ratios, which is slight in the cortex of PCG and moderate within white matter, may be due to brain post-hypoxic plasticity. The consolidation of the slow EEG activity changes over the period of prolonged observation could be connected with insufficiently efficient new neuronal networks. The wide range of different events related to this neuroplasticity could correlate not only with improvements but also with apparent deteriorations in neurological function (4, 6, 12).

## Conclusions

The authors indicate the usefulness of advanced electrophysiological and MR techniques in monitoring and long-term prognosis in patients with hypoxic brain damage. Large-scale studies on the LAS with use of the advanced electrophysiological techniques and neuroimages could help in our understanding of the rearrangement mechanisms of the damaged brain.

## Data Availability

All datasets generated for this study are included in the manuscript and/or the supplementary files.

## Author Contributions

AS made substantial contributions to acquisition of data and interpretation. ED made substantial contributions to acquisition of data and interpretation, first of all EEG. JB carried out MRI investigations. LK carried out QEEG investigation. SB made substantial contributions to conception and design and prepared the manuscript. MK carried out electrophysiological investigations and prepared the manuscript.

### Conflict of Interest Statement

The authors declare that the research was conducted in the absence of any commercial or financial relationships that could be construed as a potential conflict of interest.

## References

[1] Lance JW Adams RD. The syndrome of intention or action myoclonus as a sequel to hypoxic encephalopathy. Brain. (1963) 86:111–34. 13928398 10.1093/brain/86.1.111

[2] Shin J-H Park JM Kim AR Shin HS Lee ES Oh M-K. Lance-Adams syndrome. Ann Rehabil Med. (2012) 36:561–4. 10.5535/arm.2012.36.4.561 22977784 PMC3438425

[3] Kowalczyk EE Koszewicz MA Budrewicz SP Podemski R Slotwinski K. Lance-Adams syndrome in patient with anoxic encephalopathy in the course of bronchial asthma. Wiad Lek. (2006) 59:560–2. 17209360

[4] Gupta HV Caviness JN. Post-hypoxic Myoclonus: current concepts, neurophysiology, and treatment. Tremor Other Hyperkinet Mov. (2016) 6:409. 10.7916/D89C6XM4 27708982 PMC5039948

[5] Acharya JN Post-hypoxic myoclonus: the good, the bad and the ugly. Clin Neurophysiol Pract. (2017) 5:105–6. 10.1016/j.cnp.2017.04.002 PMC6123932 30214981

[6] Seder DB Sunde K Rubertsson S Mooney M Stammet P Riker RR. Neurologic outcomes and postresuscitation care of patients with myoclonus following cardiac arrest. Crit Care Med. (2015) 43:965–72. 10.1097/CCM.0000000000000880 25654176

[7] Pivik RT Broughton RJ Coppola R Davidson RJ Fox N Nuwer MR. Guidelines for the recording and quantitative analysis of electroencephalographic activity in research contexts. Psychophysiology. (1993) 30:547–58. 8248447 10.1111/j.1469-8986.1993.tb02081.x

[8] Venkatesan A Frucht S. Movement disorders after resuscitation from cardiac arrest. Neurol Clin. (2006) 24:123–32. 10.1016/j.ncl.2005.11.001 16443134

[9] Lee HL Lee JK. Lance-Adams syndrome. Ann Rehabil Med. (2011) 35:939–43. 10.5535/arm.2011.35.6.939 22506225 PMC3309367

[10] Richardson MP Grosse P Allen PJ Turner R Brown P. BOLD correlates of EMG spectral density in cortical myoclonus: description of method and case report. NeuroImage. (2006) 32:558–65. 10.1016/j.neuroimage.2006.04.183 16730460

[11] Zhang YX Liu JR Jiang B Liu HQ Ding MP Song SJ. Lance-Adams syndrome: a report of two cases. J Zhejiang Univ Sci B. (2007) 8:715–20. 10.1631/jzus.2007.B0715 17910113 PMC1997224

[12] Boltes Cecatto R Chadi G The importance of neuronal stimulation in central nervous system plasticity and neurorehabilitation strategies. Funct Neurol. (2007) 22:137–43 17925162

